# Systematic Review of Monocyte Transcriptomic Profiles as Diagnostic and Prognostic Biomarkers in Colorectal Cancer

**DOI:** 10.3390/ijms27094143

**Published:** 2026-05-06

**Authors:** Alicia Podadera-Herreros, Jesús Pilo, Alejandro Rego-Calvo, María Ortega-Castan, Carolina Muriel-López, Daniel Hinojosa-Nogueira, Isabel Moreno-Indias, María del Mar Amaya-Campos, Julia Alcaide-García, Hatim Boughanem, Libia Alejandra García Flores, Manuel Macías-González

**Affiliations:** 1 Department of Endocrinology and Nutrition, Virgen de la Victoria University Hospital, 29010 Malaga, Spain; 2 IBIMA Plataforma BIONAND, Instituto de Investigación Biomédica de Málaga, University of Málaga, 29590 Malaga, Spain; 3 Unidad de Gestion Clinica Cirugía General y del Aparato Digestivo, Virgen de la Victoria University, 29010 Malaga, Spain; 4 Medical Oncology Service, Hospital Regional Universitario de Málaga, 29010 Malaga, Spain; 5 CIBER Physiopathology of Obesity and Nutrition (CIBEROBN), Health Institute Carlos III (ISCIII), 28029 Madrid, Spain; 6 Department of Nutrition and Food Science, Campus of Melilla, University of Granada, 52001 Melilla, Spain; 7 Department of Cell Biology, Physiology and Immunology, University of Cordoba, 14004 Cordoba, Spain; 8 Maimonides Biomedical Research Institute of Cordoba (IMIBIC), 14004 Cordoba, Spain

**Keywords:** systematic review, monocyte transcriptomic, epitranscriptomic, colorectal cancer, gene expression profiling, personalized medicine, biomarker

## Abstract

Colorectal cancer (CRC) remains a major global health burden and a leading cause of cancer-related morbidity and mortality. Current blood-based biomarkers lack sufficient sensitivity and specificity, particularly for early detection. In this context, circulating immune-cell transcriptomic profiling has emerged as a promising minimally invasive approach. This systematic review was conducted following a PROSPERO-registered protocol (CRD42024604757) and PRISMA 2020 guidelines to evaluate the diagnostic and prognostic potential of circulating monocyte-related transcriptomic profiles in CRC. Of 295 records identified, six studies met the inclusion criteria. The available evidence consistently supports the diagnostic value of circulating transcriptomic profiles in distinguishing patients with CRC from healthy individuals and in reflecting tumour-associated immune alterations. Monocyte-related signatures, including CXCR2^+^ monocytes, were associated with disease stage and metastatic features. Epitranscriptomic modifications, such as m6A and m5C, further reinforced their diagnostic relevance, with some studies reporting higher diagnostic accuracy than classical biomarkers. In contrast, evidence for prognostic value remains limited, heterogeneous, and often indirect, largely due to small sample sizes, methodological variability, and reliance on public datasets. Overall, circulating immune-cell transcriptomic profiles are promising non-invasive biomarkers for CRC detection and characterization, although their prognostic utility remains unclear. Methodological heterogeneity limits clinical applicability, highlighting the need for standardized, CRC-specific studies with cell-type-resolved approaches.

## 1. Introduction

One of the most prevalent malignant neoplasms of the digestive system is colorectal cancer (CRC), which ranks third in incidence and mortality among malignant tumors [1]. Its incidence is increasing, particularly among younger populations and in transitioning countries, highlighting an evolving epidemiological burden. Numerous risk factors have been identified for CRC, including modifiable factors such as unhealthy lifestyle behaviors, smoking, alcohol consumption, and obesity, as well as non-modifiable factors such as age, sex, and genetic predisposition [2].

Despite the incorporation of targeted therapies, immunotherapy, and precision medicine approaches, poor prognosis and the occurrence of relapses remain prevalent, largely due to the presence of genetic and molecular heterogeneity that determines aggressiveness and sensitivity to treatments [3]. From a diagnostic perspective, colonoscopy remains the gold-standard invasive procedure; however, it requires adequate preparation, sedation, and it is often complemented by the fecal immunochemical test, which has limited sensitivity for non-bleeding lesions. Other less invasive techniques are also employed, including blood tumor biomarkers, such as cancer-secreted products (CA19–9 or CA125), substances released into the blood after tumor cell necrosis and division (CYFRA21–1), and host-reactive products of cancer (Epstein–Barr virus capsid antigen IgA) [4]. These biomarkers demonstrate limited capacity to detect early-stage disease, and their lack of diagnostic consistency is attributable to low sensitivity and specificity [5,6]. These limitations underscore the necessity for the development of sensitive and specific biomarkers, which hold great potential in enhancing the efficacy of screening and monitoring strategies.

In recent years, liquid biopsy approaches, including circulating tumor DNA and immune-cell profiling, have emerged as promising alternatives. Among these, transcriptomic technologies, primarily based on bulk RNA sequencing, enable comprehensive characterization of gene expression patterns and cellular heterogeneity [7]. Using these approaches, inflammatory phenotypes associated with worse outcomes after recurrence have been characterised, with effects varying according to stage.

Circulating immune cells, especially monocytes, represent a key component of this systemic response [8]. Monocytes are recruited to the tumor site and subsequently differentiate into tumor-associated macrophages, thereby promoting progression and metastasis [9]. Recent studies suggest that transcriptomic and epitranscriptomic alterations in these cells reflect tumor-driven systemic immune reprogramming and may serve as accessible biomarkers [10,11,12,13,14,15]. However, most of these findings are derived from bulk transcriptomic approaches, in which monocyte-related signals are captured within heterogeneous cell populations rather than being directly measured.

Therefore, this systematic review aimed to evaluate the diagnostic potential and available evidence on the prognostic relevance of circulating immune-cell transcriptomic profiles as non-invasive biomarkers in CRC, focusing on their ability to distinguish patients from healthy individuals and their association with clinicopathological features, disease progression, and tumor-related immune alterations.

## 2. Methods

### 2.1. Study Design and Protocol

This systematic review was conducted in accordance with the PROSPERO-registered protocol (CRD42024604757) and reported following the PRISMA 2020 guidelines (Supplementary Table S1) as these are standardized. [16].

This review aimed to evaluate the diagnostic potential and available evidence on the prognostic relevance of circulating monocyte transcriptomic profiles in CRC, including their association with clinicopathological features, disease progression, and survival outcomes (overall survival (OS), disease-free survival (DFS), and cancer-specific survival (CSS), as specified in the registered protocol.

### 2.2. Data Sources and Search Strategies

A systematic literature search was conducted in PubMed, Scopus, Web of Science, and Embase to identify relevant studies published between 1 January 2019 and 31 December 2024, in accordance with the registered protocol (PROSPERO CRD42024604757).

The search strategy was developed according to the PICOS framework and combined controlled vocabulary and free-text terms related to CRC, circulating monocytes, transcriptomic profiling, biomarkers, and clinical outcomes. Given that the primary population of interest was circulating monocytes, this was specifically prioritized in the search terms and study selection. However, considering the widespread use of bulk transcriptomic approaches, studies reporting data from whole blood or peripheral blood mononuclear cells (PBMCs) were also included when relevant, as these may capture monocyte-related signals within mixed-cell populations. Database-specific adaptations were applied where necessary. The full search strategy for each database is provided in Supplementary Table S2.

The search was restricted to studies published in English and to primary research articles with original data. Reviews, meta-analyses, conference proceedings, and studies without original data were excluded. Eligibility criteria were predefined according to the PICOS framework (Table 1). The literature search was repeated prior to the final analysis to ensure the inclusion of any additional eligible studies.

### 2.3. Study Selection and Data Extraction

Three reviewers (A.P., D.H., and J.P.) independently screened studies in two stages: initial screening of titles and abstracts, followed by full-text assessment against predefined eligibility criteria. The study selection process was conducted using the CADIMA systematic review platform to ensure a structured and transparent workflow. Discrepancies at any stage were resolved through discussion and, when necessary, consultation with a fourth reviewer (L.A.G.-F.). Only studies meeting the predefined inclusion criteria and providing original data were included in the final analysis.

Data extraction was performed using the CADIMA platform by two independent reviewers (A.P. and D.H.), with verification by a third reviewer to ensure consistency and accuracy. Any discrepancies were resolved through discussion or adjudication by a third reviewer (L.A.G.-F.). Data extraction was performed using a standardized form and included study characteristics (authors, year, country, and design), population characteristics (age, sex, and baseline features), transcriptomic profiling methods, clinical and survival outcomes (e.g., OS, DFS, CSS), as well as CRC classification, and relevant tumor, inflammatory, and immune-related biomarkers. For studies including external validation datasets (e.g., GEO or TCGA), these were analyzed within the context of the original study and were not treated as independent units to avoid duplication bias.

### 2.4. Risk-of-Bias Assessment

Risk of bias was assessed using the CADIMA systematic review platform across four predefined domains (D1–D4), with qualitative ratings assigned as low, some concerns, high, or unclear according to predefined criteria. A domain-based approach implemented in CADIMA was applied to ensure a consistent assessment. CADIMA includes a dedicated section for critical appraisal, defined as the systematic evaluation of the methodological quality and potential risk of bias of included studies. In this section, studies are assessed across predefined domains (e.g., selection bias, measurement of outcomes, and reporting bias) using structured signaling questions, allowing each domain to be rated and an overall judgment to be assigned. Within this framework, observational studies and analyses derived from public transcriptomic databases were treated as separate data sources, and the risk of bias was assessed independently for each study type to account for their distinct methodological characteristics.

An overall assessment was derived using a conservative hierarchical approach: studies were classified as high risk if at least one domain was rated high; as some concerns if no domains were rated high but at least one showed some concerns; and as low risk only if all domains were rated low. Studies were classified as unclear when insufficient information precluded a definitive assessment. Data were exported from CADIMA and analyzed in R (version 4.5.2) using the dplyr, tidyr, and ggplot2 packages. Risk-of-bias distributions were summarized descriptively and visualized using customized ROBVIS-style plots. This approach allowed a standardized and transparent evaluation of methodological quality despite variability in study design, data sources, and transcriptomic methodologies.

## 3. Results

### 3.1. Literature Search

**Figure 1:**
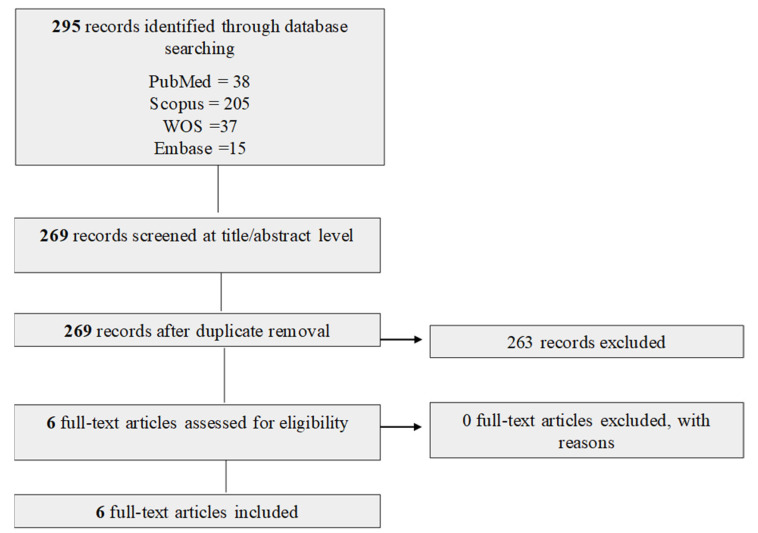
PRISMA flow diagram for the systematic review. A total of 295 records were added to CADIMA after all database searches were concluded. After duplicate removal, 263 were screened by title and abstract, and 6 articles remained for full-text screening [10,11,12,13,14,15].

A total of 295 records were identified through database searches (PubMed, Scopus, Web of Science, and Embase) and imported into CADIMA. After removal of duplicates, 263 records were screened based on titles and abstracts. Of these, six studies met the eligibility criteria and were included in the final analysis. The study selection process is summarized in the PRISMA flowchart diagram (Figure 1).

### 3.2. Study Characteristics

Descriptive characteristics of the included studies (*n* = 6) are presented in Table 2. All studies were published between 2019 and 2024 and employed observational designs, including four prospective and two retrospective studies.

Overall, the study populations were heterogeneous, particularly in demographic reporting. A predominance of male participants was observed across most studies, although sex distribution was not consistently reported. Age was presented as mean or median values, ranging from 61.5 to 63 years in two studies [10,12], and two studies categorized age (<60 vs. ≥60 years), both showing a higher proportion of participants younger than 60 years [14,15]. In one study, all patients were older than 50 years [11], whereas age distribution was not reported in one study [13]. Geographically, four studies were carried out in China [12,13,14,15], one in the United States [10], and one in South Korea and Switzerland [11]. All studies included patients diagnosed with stage I–III CRC.

From a methodological perspective, most studies relied on publicly available transcriptomic datasets, typically using a discovery–validation framework (Table 2). In these cases, the primary population within each study was considered the discovery cohort, while additional datasets were classified as validation cohorts. External validation was commonly performed using Gene Expression Omnibus (GEO) datasets across most studies, including Martinez et al. [11] (GSE156451, GSE196006, GSE89393, GSE109203, GSE136630, GSE76987 and GSE164541 from China, United States and Poland), Xie et al. [14] (GSE164191 from China), Yin et al. [15] (GSE10715 from Hungary), Wang et al. [12] (GSE47756 from Belgium), and Dyikanov et al. [10] (GSE201085 from the United States). In addition, one study integrated multiple public databases, including TCGA-COAD, CCLE, Tumor, TISIDB, and TIMER databases [13].

Transcriptomic profiling approaches varied across studies (Table 2). Xie et al. and Yin et al. quantified epitranscriptomic modifications using colorimetric and fluorometric assays, followed by validation through microarray-based expression profiling in external datasets [14,15]. Wu et al. integrated data from public repositories, including TCGA-COAD and the Human Colon Cancer Atlas, analyzing cancerous and non-cancerous tissues using bulk RNA-seq and single-cell RNA-seq, respectively [13]. Martinez et al. and Dyikanov et al. performed transcriptomic analyses using bulk RNA-seq [10,11]. In contrast, one study quantified target mRNA expression using quantitative RT-PCR rather than RNA-seq [12].

Regarding cancer type and diagnostic context, the included studies primarily focused on patients diagnosed with CRC prior to surgical intervention or adjuvant treatment, as summarized in Table 2. In most cases, the main study populations consisted of treatment-naïve patients, complemented by external validation datasets [11,12,13,14,15]. Only one study reported a follow-up period of 14 days after surgery [14]. In contrast, Dyikanov et al. included a broader cohort of 84 solid tumors, among which CRC was represented, with 50.6% of patients having received prior treatment, including chemotherapy, radiotherapy, immune checkpoint inhibitors, or other systemic therapies [10]. Moreover, the external validation datasets in this study included tumor types other than CRC, introducing additional heterogeneity in cancer type and treatment exposure. These characteristics highlight the heterogeneity in study design, populations, and transcriptomic methodologies, which should be taken into account when interpreting the findings.

### 3.3. Transcriptomic–Clinical Associations in CRC

Considering the heterogeneity across studies, associations between monocyte-related transcriptomic profiles and clinical outcomes in CRC were variable and context-dependent.

Overall, the available evidence supports the evaluation of circulating transcriptomic profiles from both diagnostic and prognostic perspectives in CRC. In this section, we first assess their diagnostic value and associations with clinicopathological features. As summarized in Table 3, several transcriptomic signatures showed limited or no clear association with conventional tumor biomarkers such as CEA, CA125, or CA19–9, while in some cases demonstrating improved diagnostic performance compared to these markers. These findings support their potential role in CRC detection and classification. Wang et al. reported that CXCR2^+^ monocyte levels were not associated with conventional tumor markers, including CEA, CA-125, CA19–9, and CA72–4. However, they exhibited higher sensitivity than CEA in distinguishing intermediate stages (II–III) and were significantly increased in patients without nodal or metastatic involvement (N0/M0) compared to those with lymph node or distant metastasis (N1–N2 or M1–M2), suggesting their potential as stage-associated biomarkers of disease progression. Notably, epitranscriptomic modifications (m6A and m5C) in peripheral blood mononuclear cells were associated with tumor progression, metastatic status, and, in some cases, showed higher diagnostic accuracy than classical biomarkers [14,15]. Additionally, specific monocyte-related signatures, such as CXCR2^+^ monocytes, were linked to tumor stage and metastatic spread, showing higher sensitivity than CEA in distinguishing intermediate stages. Other transcriptomic markers, including FABP4 expression, were consistently downregulated in tumor tissues, although their direct association with classical biomarkers was not always reported [13]. Martínez et al. [11] reported a panel of 524 candidate genes capable of discriminating patients with CRC from healthy controls. Notably, early stages were characterized by activation of innate immune responses, followed by transient B-cell activation and progressive suppression of T-cell activity, while advanced stages showed enrichment of protumoral myeloid signatures, wound healing, and coagulation pathways. These findings were consistently validated across independent datasets, supporting the robustness of peripheral transcriptomic alterations as reflections of tumor progression. Importantly, the parallel alterations observed between tumor tissue and PBMCs suggest that systemic immune modulation mirrors local tumor biology, even at early stages.

Subsequently, we evaluated the prognostic perspective, focusing on associations with survival outcomes (OS, DFS, CSS) and treatment response, for which the available evidence was comparatively more limited and less consistent across studies. For instance, Wu et al. reported survival-related findings; however, these were primarily associated with tumor tissues rather than circulating immune cells, based on data from the TCGA-COAD database [13]. Similarly, Dyikanov et al. described associations with survival outcomes, although these analyses were derived from validation cohorts in other cancer types, including breast cancer, pancreatic ductal adenocarcinoma, and head and neck squamous cell carcinoma, thereby limiting their direct applicability to CRC [10].

From an immunological perspective, transcriptomic alterations were consistently linked to immune-related processes (Table 3). Monocytes emerged as a key immune cell population, particularly in relation to epitranscriptomic modifications (m6A and m5C), which were associated with monocyte infiltration, chemotaxis, and cytokine production. Several studies reported interactions between transcriptomic signatures and both innate and adaptive immune responses. Martínez et al. [11] identified that early stages were characterized by activation of innate immune responses, followed by transient B-cell activation and progressive suppression of T-cell activity, while advanced stages showed enrichment of protumoral myeloid signatures, wound healing, and coagulation pathways. Furthermore, specific immune profiles were associated with inflammatory mediators such as IL-6, IFN-γ, and TNF-α as well as with tumor-associated macrophage polarization and systemic inflammatory markers [12]. Functional enrichment analyses consistently highlighted pathways related to immune activation, antigen presentation, wound healing, and myeloid cell activation, supporting a central role of immune dysregulation in CRC-associated transcriptomic patterns.

### 3.4. Risk of Bias

**Figure 2:**
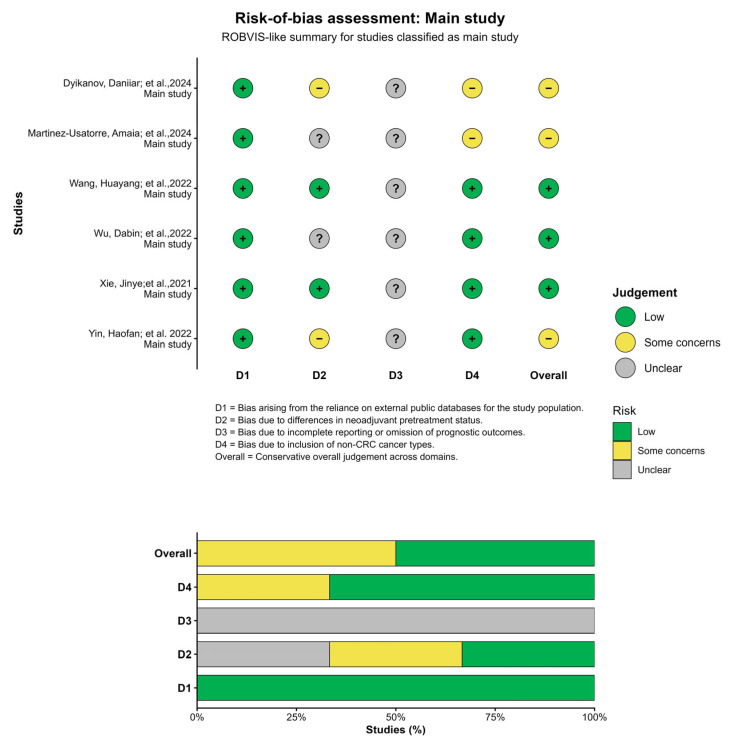
Risk-of-bias assessment of included main studies [10,11,12,13,14,15]. Risk of bias was evaluated across four predefined domains (D1–D4) and summarized as an overall judgment for each study. The upper panel shows individual study-level assessments, where each symbol represents the judgment per domain (green: low risk; yellow: some concerns; grey: unclear). The lower panel presents the distribution of risk-of-bias judgments across studies for each domain and overall.

The risk-of-bias assessment across the included main studies showed heterogeneity across domains (Figure 2). Most studies were rated as low risk in D1, reflecting appropriate use of transcriptomic data sources. In contrast, greater variability was observed in D2 (neoadjuvant pretreatment differences) and D4 (inclusion of non-CRC cancer types), with several studies classified as having some concerns. Domain D3 (incomplete reporting of prognostic outcomes) was predominantly rated as unclear due to insufficient information.

Overall, the majority of studies were classified as low risk or some concerns, with no studies reaching an overall high-risk classification. However, several studies showed incomplete or inconsistent reporting of survival outcomes (OS, DFS, CSS), as well as substantial variability in patient populations, including differences in disease stage and treatment status (Figure 2).

In contrast, the risk-of-bias assessment of external datasets (including database studies and validation cohorts) revealed a predominance of high-risk classifications across several domains (Supplementary Figure S1). Domain D1 showed the highest proportion of studies at high risk of bias, reflecting inherent limitations related to study population definition and data sourcing. Domain D2 demonstrated substantial heterogeneity, with a mix of high-risk, low-risk, and unclear classifications. Domain D3 was largely rated as unclear, indicating insufficient reporting across studies. In contrast, D4 showed a more balanced distribution, with a considerable proportion of studies classified as low risk. Overall, external datasets were more frequently classified as high risk, highlighting additional methodological limitations associated with secondary data sources and reinforcing the overall heterogeneity of the evidence.

## 4. Discussion

Colorectal cancer remains a major global health burden, with increasing incidence rates, particularly among younger populations, highlighting the need for improved non-invasive diagnostic and prognostic strategies. In this context, transcriptomic profiling has emerged as a powerful approach to capture dynamic molecular changes associated with tumor development and immune system activity.

To further explore the diagnostic utility of circulating transcriptomic profiles, several studies have identified that cancer alters circulating monocytic populations and their transcriptomes, findings that have also been validated in animal and in vitro models [9,17,18,19]; however, their characterization in patients with CRC lacks comprehensive characterization. In this systematic review, distinct transcriptomic alterations in peripheral blood were associated with disease, demonstrating the ability to differentiate CRC patients from healthy controls across different stages and showing consistent potential as diagnostic biomarkers. Collectively, these studies underscore two complementary strategies: the targeted validation of immune cell–specific biomarkers [12,13] and the unbiased exploration of global transcriptomic signatures [10,11,14,15]. In line with this, not only transcriptomic but also epitranscriptomic profiles are altered in CRC. Since the 2011 discovery that m6A is a reversible modification [20], the field of epitranscriptomics has expanded dramatically [21,22], leveraging this dynamic property to overcome drug resistance in cancer [23]. Studies such as those by Xie et al. and Yin et al. show that epitranscriptomic modification levels, including m5C and m6A, are altered in peripheral blood immune cells from patients with CRC, demonstrating higher diagnostic accuracy than classical tumor markers [14,15]. Together, these studies indicate that both transcriptomic and epitranscriptomic profiling of circulating immune cells may serve as non-invasive tools for CRC detection.

To address the prognostic utility of circulating transcriptomic profiles, the available evidence remains limited and heterogeneous. As observed in the Results section, survival associations were often derived from tumor tissue analyses or from validation cohorts including other cancer types, thereby limiting their direct applicability to CRC. Wu et al. reported survival-related findings with FABP4-related immunomodulators, a fatty acid–binding protein mainly expressed in adipose tissue and macrophages [24]; however, these findings were mainly linked to cancerous tissues rather than circulating immune cells. Similarly, reduced FABP4 expression in tumor tissues has been reported in CRC [25], although its role in circulation remains unclear. In breast cancer cell models, elevated exogenous FABP4 may promote tumor progression by enhancing proliferation, upregulating FoxM1, and increasing nutrient supply and adipokine signaling through CD36 and FABP5 expression [26]. Therefore, the role of circulating FABP4, particularly in CRC, as well as its differential transcriptional levels, warrants further investigation. Interestingly, Djikanov et al. did not directly associate transcriptomic profiles with survival outcomes or classical clinicopathological variables such as TNM stage or CEA levels, as their unsupervised approach focused on defining novel immune-based clusters (immunotypes), primarily related to treatment response [10]. Although survival associations were reported, these analyses were ultimately derived from validation cohorts in other cancer types, including breast cancer, pancreatic ductal adenocarcinoma, and head and neck squamous cell carcinoma, rather than CRC specifically. This highlights a critical gap in the current literature and underscores the need for studies specifically designed to evaluate the prognostic relevance of circulating transcriptomic biomarkers in CRC. In particular, CRC prognosis may be influenced by challenges in early-stage diagnosis, driven by the high cost and invasiveness of colonoscopy and the limited sensitivity of circulating biomarkers, which may contribute partially to the lack of long-term prognostic assessment [1,5,27]. Consequently, a substantial proportion of patients are still diagnosed at advanced stages, with approximately 20% presenting with metastatic disease at diagnosis [28]. Moreover, tumor heterogeneity may also contribute to the limited and inconsistent prognostic evidence, as patients within the same stage can exhibit markedly different survival outcomes [29,30]. In addition to the intrinsic heterogeneity of cancer, location-dependent differences between colon and rectal tumors have also been described. Specifically, in colon but not rectal patients, monocytes display a transcriptional program characterized by enhanced activation of glycolytic pathways, which has been associated with a pro-tumoral phenotype of monocyte-derived tumor-associated macrophages (TAMs) and poorer clinical outcomes [31]. In this context, future studies should consider strategies to overcome tumor heterogeneity and anatomical location to identify more consistent molecular classifications with improved prognostic relevance [7,32,33].

From a biological perspective, the included studies consistently pointed to immune dysregulation as a central feature of CRC, with monocytes playing a key role in tumor-associated processes. According to the literature, monocyte counts have been identified as an independent prognostic factor for predicting the outcome in CRC patients [34,35]. However, although the search strategy specifically targeted circulating monocytes, it also retrieved studies based on whole blood or peripheral blood mononuclear cells, due to the widespread use of bulk transcriptomic approaches that capture monocyte-related signals within heterogeneous cell populations rather than directly measuring isolated monocytes. Three studies specifically highlighted the relevance of monocyte populations. For instance, Xie et al. and Yin et al. identified monocytes as the immune cells most strongly associated with the levels of epitranscriptomic modifications [14,15], while Wang et al. directly demonstrated their role, particularly their transition into tumor-associated macrophages within the tumor microenvironment [12]. However, Martínez et al. identified neutrophils as the most prominent contributors to the observed transcriptomic changes, although a role for monocytes was also observed [11].

Taken together, these findings suggest that circulating transcriptomic biomarkers may complement existing screening and monitoring strategies, particularly in early-stage disease or in settings where minimally invasive approaches are required. However, their prognostic relevance remains insufficiently established due to the limited number of available studies and methodological and biological heterogeneity, introducing variability in resolution, data processing, and cohort composition. Moreover, there is a lack of large, well-characterized CRC-specific cohorts with standardized approaches. Future research should prioritize well-designed, prospective, CRC-specific longitudinal studies with comprehensive clinical annotation. Additionally, methodological heterogeneity, compounded by the intrinsic cellular heterogeneity of tumors, remains a major challenge for the interpretation of transcriptomic findings. Future approaches should incorporate higher-resolution and integrative strategies, including single-cell and single-nucleus RNA sequencing, spatial transcriptomics, and computational deconvolution of bulk data, to better resolve cell-type–specific signals. The combination of these methodologies, together with harmonized experimental and analytical frameworks and standardized monocyte isolation strategies, will be essential to improve reproducibility, enhance cell-type specificity, and facilitate the clinical translation of transcriptomic biomarkers in CRC.

## 5. Conclusions

Circulating immune-cell transcriptomic and epitranscriptomic profiles show consistent potential as non-invasive diagnostic biomarkers in CRC, particularly in relation to tumour stage and disease-related immune alterations. However, current evidence supporting their prognostic value remains limited, heterogeneous, and often indirect, precluding robust CRC-specific conclusions. In addition, substantial methodological and biological heterogeneity across studies, including differences in transcriptomic platforms, patient populations, and the use of mixed-cell populations, represents a major limitation for interpretation and clinical translation. Future research should prioritize well-designed, CRC-specific longitudinal studies with standardized methodologies, including cell-type–specific approaches such as monocyte isolation strategies, to improve reproducibility and enable more precise biological and clinical interpretation.

## Figures and Tables

**Table 1:** PICOS criteria for inclusion and exclusion of studies.

Criteria	Inclusion Criteria	Exclusion Criteria
Population	- Adults (18+ years) diagnosed with colorectal cancer (CRC) - Both male and female patients - Patients across all stages of colorectal cancer (Stage I–IV)	- Pediatric or adolescent populations - Pregnant or breastfeeding women - Patients with significant co-morbidities unrelated to CRC (e.g., autoimmune disorders, other cancers) that could confound results.
Intervention/Exposure	- Transcriptomic Profiling: Studies that perform transcriptomic analysis on circulating monocytes in colorectal cancer (CRC) patients, using techniques such as RNA sequencing (RNA-Seq), microarrays, or other high-throughput technologies. - Monocyte Isolation: Studies where monocytes are isolated from peripheral blood samples using standardized methods such as Ficoll–Paque density gradient centrifugation, magnetic bead separation (e.g., CD14+ selection), or flow cytometry, ensuring that the purity and integrity of monocytes are maintained for accurate transcriptomic analysis. - Focus on Circulating Monocytes: Studies specifically focusing on the transcriptomic profiles of circulating monocytes as opposed to other immune cells or tissue-resident monocytes/macrophages. - Timing of Sample Collection: Studies that specify the timing of blood sample collection, particularly preoperative, postoperative, or at different stages of CRC progression, to assess how surgical intervention and disease stage impact monocyte transcriptomics.	- Non-Transcriptomic Studies: Studies that do not involve transcriptomic profiling (e.g., proteomic or metabolomic studies), or that focus on gene expression at the DNA or protein level without transcriptomic data. - Non-Monocyte Studies: Studies focusing on other immune cells (e.g., T-cells, B-cells, macrophages) or non-circulating monocytes (e.g., tissue-resident macrophages in the tumor microenvironment). - Unclear Methodology: Studies that do not clearly describe the methods used for monocyte isolation or transcriptomic analysis, leading to potential concerns about the accuracy and reliability of the data. - Animal or In Vitro Studies: Studies conducted solely in animal models or in vitro (cell culture) systems, as the focus of this review is on human clinical data.
Comparator	- Healthy controls - CRC patients at different cancer stages (Stage I–IV)	- Studies comparing non-relevant groups (e.g., chemotherapy vs. radiotherapy) - Studies without clear control or comparator groups
Outcomes	- Prognostic outcomes (e.g., overall survival [OS], disease-free survival [DFS], cancer-specific survival [CSS]) - Correlations with clinicopathological features (e.g., tumor stage, CEA levels, tumor size, tumor location, depth of invasion, presence of metastasis)	- Studies without relevant outcome data - Studies focusing on non-transcriptomic biomarkers - Studies that do not report on survival or clinicopathological correlations.
Study Design	Observational studies (e.g., prospective and retrospective cohort studies).	- Case reports

Abbreviations: B-cells, B lymphocytes; CD14+, cluster of differentiation 14 positive cells; CEA, carcinoembryonic antigen; CRC, colorectal cancer; CSS, cancer-specific survival; DFS, disease-free survival; DNA, deoxyribonucleic acid; OS, overall survival; RNA-Seq, RNA sequencing; T-cells, T lymphocytes.

**Table 2:** Design, population, and characteristics of the studies.

Reference	Study Design	Population	Country	Age (Years)	Sex Distribution (*n*, Female/Male)	Basal Characteristics	Diagnosis Details	Transcriptomic Profiling Method
[13]	Prospective study	Discovery set 10 patients with colon adenocarcinoma (cancerous and noncancerous tissue)	China	NR	NR	NR	Patients diagnosed with colon adenocarcinoma and received primary surgery.	Immunochemistry
	Retrospective study	TCGA-COAD database - Cancerous tissue = 473 - Noncancerous tissues = 41	Several	NR	NR	NR	From public TCGA-COAD database: Patients diagnosed with colon adenocarcinoma	Bulk RNA-Seq
	Retrospective study	TIMER database From Human Colon Cancer Atlas (c295) in Single Cell Portal. - Cancerous tissue = 28 - Noncancerous tissues = 34	Several	NR	NR	NR	From public GEO database. Patients diagnosed with CRC	Single-cell RNA-seq
	Retrospective study	CCLE and TISIDB database	Several	NA	NA	NA	Human cancer cell lines	NA
[12]	Prospective study	Discovery set PBMCs from CRC patients = 101	China	62.0 ± 12.1 (mean ± SD)	101, 31/70	Location Rectum = 53 Rectosigmoid = 10 Colon = 38 TNM Stage I = 8 II = 28 III = 43 IV = 12 Growth Pattern Infiltrative = 64 Exophytic = 25 Censored = 12 Histology grade Poorly differentiated = 22 Moderately differentiated = 59 Well differentiated = 16 Censored = 4 Pre-operative tumor markers (ng/mL) CEA = 9.1 ± 12.2 CA−199 = 21 ± 25.8 CA-125 = 16.5 ± 33.1 CA-724 = 6.3 ± 12.2	Patients diagnosed with colorectal cancer and received primary surgery. None of the patients had received preoperative radiotherapy/chemotherapy or were diagnosed with other types of primary tumors	Quantitative RT-PCR
	Retrospective study	GSE47756 - Healthy PBMCs = 38 - Non-metastatic PBMCs CRC = 27 - Metastatic PBMCs CRC = 28	Belgium	NR	NR	NR	From public GEO database. Patients sporadic histologically confirmed adenocarcinoma of the colon and/or rectum diagnosed: 27 patients with non-metastatic stage I, stage II or stage III CRC, 28 patients with metastatic stage IV CRC, and 38 healthy volunteers (without history or evidence of acute or chronic disease). All patient samples were collected after histological diagnosis upon screening colonoscopy, prior to any treatment.	Expression profiling by array
[14]	Retrospective study	Discovery set - Healthy PBMCs = 64 - PBMCs CRC = 105	China	Age ≤ 60 years = 57 Age > 60 years = 48	105 (36,69)	Clinical stage I = 6 II = 20 III = 31 IV = 26 T classification T1–T2 = 15 T3–T4 = 64 *n* classification N0 = 29 N1–N2 = 50 *n* classification N0–N1 = 57 N2 = 22 M classification M0 = 57 M1 = 26 Differentiation Poor = 14 Moderate/Well = 70 Tumor budding Bd1–Bd2 = 12 Bd3 = 16 HER2 expression Negative = 26 Positive = 26 KRAS genotyping Wild type = 10 Mutation type = 7 BRAF genotyping Wild type = 17 Mutation type = 3 CEA (ng/mL) <5 = 44 ≥5 = 54 CA125 (ng/mL) <35 = 68 ≥35 = 30 CA19–9 (ng/mL) <35 = 66 ≥35 = 32	Patients diagnosed with colorectal cancer: 105 CRC patients and 64 healthy individuals who had no history of basic or chronic diseases. All CRC patients were diagnosed on the basis of the histopathology by biopsy or endoscopic examination, before surgery or radiochemotherapy. In 33 CRC patients peripheral blood was also collected 14 days after surgery.	m6A levels by colorimetric assay.
	Retrospective study	GSE164191 - Healthy PBMC individuals = 62 - PBMCs CRC = 59	China	Healthy individuals 51.6 ± 5.5 CRC patients 63.0 ± 11.4	Healthy individuals 62 (24/38) CRC patients 59 (21/38)	Tumor location Left colon = 14 Right colon = 7 Rectum = 34 Unknown = 4 Tumor differentiation Well = 2 Well-moderate = 1 Moderate = 42 Moderate-Poor = 4 Poor = 4 Unknown = 6 Pathological Tumor-Node-Metastasis stage I = 7 II = 22 III = 27 IV = 1 Unknown = 2	From public GEO database. Patients diagnosed during a routine colonoscopy with CRC: 59 patients with CRC before any form of treatment, including radio- and chemotherapy or surgery.	Expression profiling by array
[15]	Retrospective study	Discovery set Training set - Healthy PBMC individuals = 53 - PBMCs CRC = 134 Validation set - Healthy PBMC individuals = 30 - PBMCs CRC = 62	China	Training set Age ≤ 60 years = 50 Age > 60 years = 42	Training set 92 (34/58)	Clinical stage I–II = 30 III–IV = 62 T classification T1–T2 = 32 T3–T4 = 60 *n* classification N0 = 30 N1–N2 = 62 M classification M0 = 62 M1 = 30 Differentiation Poor = 13 Moderate/Well = 79 Tumor budding Bd1–Bd2 = 11 Bd3 = 16 HER2 expression Negative = 26 Positive = 22 KRAS genotyping Wild-type = 8 Mutation-type = 8 BRAF genotyping Wild-type = 15 Mutation-type = 3 CEA (ng/mL) <5 = 54 ≥5 = 38 CA125 (ng/mL) <35 = 70 ≥35 = 22 CA19–9 (ng/mL) <35 = 68 ≥35 = 24	Training set Patients diagnosed with CRC: 134 CRC patients (92 CRC patients were collected when initially diagnosed before surgery or radiochemotherapy and 42 CRC patients had already received treatment at the time of sample collection) and 53 healthy individuals who had no history of basic or chronic diseases were collected from the Zhongshan People’s Hospital Validation set 62 CRC patients and 30 HC were collected from the Sun Yat-sen University Cancer Center as the validation set.	m5C levels by fluorometric assay.
	Retrospective study	GSE10715: - Healthy individuals = 11 - CRC = 19	Hungary	NR	NR	NR	From public GEO database. Patients diagnosed with colorectal cancer: 19 colorectal cancer and 11 healthy patients.	Expression profiling by array
[11]	Prospective study	Peripheral blood samples from: Discovery set (*n* = 295) - Advanced PBMCs adenoma = 64 - PBMCs CRC = 85 - Control PBMCs = 108 - Other PBMCs cancers = 38 Validation set (*n* = 275) - Advanced PBMCs adenoma = 50 - PBMCs CRC = 109 - PBMCs Control = 116	South Korea and Switzerland	Individuals older than 50 years	NR	NR	1665 subjects >50 years undergoing colonoscopy/surgery; 570 PBMC samples analyzed. Blood collected −30 days to +12 weeks from colonoscopy, prior to surgery. Sequencing batches: discovery (*n* = 295; batches 1–2) and validation (*n* = 275; batch 3). Population: controls (no lesions/hyperplastic polyps), advanced adenoma (AA; no/low/high dysplasia), CRC (stages I–IV), and other cancers (lung, prostate, pancreas).	Bulk RNA-Seq
	Retrospective study	GSE164541 CRC, adenoma and normal adjacent tissue = 5 patients with CRC.	China	NR	NR	NR	From public GEO database.	Bulk RNA-Seq
	Retrospective study	GSE196006, GSE89393, GSE109203, GSE136630, GSE76987 and GSE164541	China, United States and Poland.	NR	NR	NR	From the public GEO database, six additional independent CRC tumor RNA-seq datasets were included, yielding a total of seven datasets comprising 119 tumor samples and 132 normal tissues from three different countries.	Bulk RNA-Seq
[10]	Prospective study	Discovery cohort Peripheral blood from 408 healthy donors and 442 cancer patients, aged 16 to 98 years (*n* = 850, internal cohort)	United States	Healthy donors = 47 years Cancer patients = 61.5 years	NR	NR	This cohort comprised 84 different solid tumor diagnoses (pancreatic cancer (*n* = 37), breast neoplasm (*n* = 65), non-small cell lung carcinoma (*n* = 32), colorectal neoplasm (*n* = 41), melanoma (*n* = 19), and prostate cancer (*n* = 18) within seven major therapy groups. A total of 211 patients (211/417, 50.6%) underwent previous treatments within a year of blood draw, including chemotherapy, radiotherapy, immune checkpoint inhibitor (ICI), or other types of systemic therapy. A total of 234 patients (234/417, 56.1%) were on ongoing therapy during material collection, while 44 patients (44/417, 10.55%) had no evidence of therapy administration after cancer diagnosis.	Bulk RNA-Seq
	Retrospective study	GSE201085	United States	NR	NR	NR	Patients diagnosed with breast cancer: 30 days after being treated with neoadjuvant chemotherapy (NAC) prior to surgical resection.	Bulk RNA-Seq
	Retrospective study	PDAC (randomized phase II clinical trial (PRINC)	United States	≥65 y = 13% (37)	35, (16/19)	NR	These patients were treated with a combination of chemotherapy and anti-PD-1 (nivolumab) or anti-CD40L (sotigalimab) immunotherapy, or all three. PBMCs collected 30 days after treatment	Bulk RNA-Seq
	Retrospective study	HNSCC HNSCC-Nivo: 35 HNSCC patients (HNSCC-Nivo cohort) treated with first-line nivolumab alone or nivolumab in combination with indoleamine 2,3dioxygenase-1 inhibitor BMS-986205 (IDOi HNSCC-Durva: all 32 HNSCC patients (HNSCC-Durva cohort) were treated with PD-L1 inhibitor durvalumab; among these, 25 were also treated with antihyperglycemic agent metformin.	HNSCC-Nivo: United States HNSCC-Durva: United States	HNSCC-Durva: 60.781 (±11.499) HNSCC-Nivo: 63.486 ± 9.443	HNSCC-Durva: 32 (9/23) HNSCC-Nivo: 35 (5/29)	HNSCC-Durva Treatment response: NR = 14 R = 18 MIS pretherapy G1-naïve = 5 G2-Primed = 10 G3-Progressive = 7 G4-Chronic = 4 G5-Suppressive = 5 no data = 1 HPV status Negative = 13 Positive = 19 HNSCC-Nivo: Therapy Nivolumab = 10 Nivolumab + IDO = 25 Treatment Response NR = 15 R = 20 MIS pretherapy G1_Naive = 6 G2_Primed = 8 G3_Progressive = 8 G4_Chronic = 5 G5_Suppressive= 8 HPV status Negative = 17 Positive = 18	HNSCC-Nivo: patients received anti-PD-1 monoclonal antibody nivolumab or nivolumab plus a specific IDO inhibitor HNSCC Durva cohort: anti-PDL1 monoclonal antibody durvalumab or durvalumab plus metformin treated	Bulk RNA-Seq

Overview of the design, population, and main characteristics of the six selected studies, including study type, sample size, patient demographics, biological sources, and key methodological approaches [10,11,12,13,14,15]. NR, Not reported; NA, Not Applicable. Abbreviations: AA, advanced adenoma; BMS, Bristol-Myers Squibb; CA-125, cancer antigen 125; CA-199, cancer antigen 19–9; CA-724, cancer antigen 72–4; CEA, carcinoembryonic antigen; CCLE, Cancer Cell Line Encyclopedia; CRC, colorectal cancer; GEO, Gene Expression Omnibus; HER2, human epidermal growth factor receptor 2; HNSCC, head and neck squamous cell carcinoma; HPV, human papillomavirus; ICI, immune checkpoint inhibitor; IDO, indoleamine 2,3-dioxygenase; m5C, 5-methylcytosine; m6A, N6-methyladenosine; NAC, neoadjuvant chemotherapy; NR, not reported; PBMCs, peripheral blood mononuclear cells; PD-1, programmed cell death protein 1; PD-L1, programmed death-ligand 1; PDAC, pancreatic ductal adenocarcinoma; RNA-Seq, RNA sequencing; TCGA-COAD, The Cancer Genome Atlas Colon Adenocarcinoma; TIMER, Tumor Immune Estimation Resource; TISIDB, Tumor–Immune System Interaction Database; TNM, tumor–node–metastasis.

**Table 3:** Association of transcriptomic profile with CRC classification, classical tumour biomarkers and inflammatory cells.

Reference	Prognostic Value in Relation to Survival Outcomes (OS, DFS, CSS) and Treatment Response	Diagnostic Value in CRC Classification and Association with Classical Tumor Biomarkers	Inflammatory Markers and Immune Cell Profile
[13] Discovery set	NR	NR	↓ FABP4 protein level in cancerous tissues by immunohistochemistry
TCGA-COAD database	1- and 3-year OS prediction in COAD based on nomogram (FABP4-related immunomodulators, stage, age, sex; C-index = 0.584).	↓ FABP4 mRNA expression in cancerous tissues.	NR
TIMER database	NR	NR	FABP4 mRNA expression was associated with B cells, CD4^+^ T cells, CD8^+^ T cells, myeloid dendritic cells, macrophages, and neutrophils.
CCLE and TISIDB	NR	2-gene FABP4-related immunomodulator COAD risk score (*p* = 0.023); ROC-AUC = 0.802 (with clinical variables).	54 immunomodulators correlated with FABP4 in COAD. FABP4 co-expressed genes enriched in immune-related pathways: innate immune response, defense response and antigen processing/presentation.
[12] Discovery set	NR	No association CXCR2^+^ monocytes levels with CEA, CA-125, CA19–9, CA72–4. ↑ sensitivity than CEA for distinguishing stage II–III. ↑ CXCR2^+^ monocytes levels in N0/M0 vs. N1–N2 or M1–M2.	CXCR2^+^ monocytes inversely associated with systemic inflammation (CRP, ESR, mGPS) and positively associated with local tumor inflammatory score and CD68^+^ tumor-infiltrating macrophage ↑ IL-6, IFN-γ, and TNF-α in CXCR2-low patients: IL-6 (10.72 ± 7.65 vs. 6.14 ± 2.10 pg/mL), IFN-γ (78.27 ± 21.49 vs. 60.27 ± 17.31 pg/mL), and TNF-α (8.19 ± 2.30 vs. 12.38 ± 10.45 pg/mL). No difference in IL-1β, IL-4, IL-8, and IL-10. CRC-derived TAMs showed ↓CD86/HLA-DR; ↑CD163/CD206; ↑IL-6, IL-10, CCL2; ↓IL-1β.
GSE47756	NR	No differences in CXCR2 mRNA in PBMCs across healthy, non-metastatic, and metastatic CRC.	GO enrichment in inflammatory/immune pathways.
[14] Discovery set	NR	↑ m6A levels in CRC PBMCs vs. controls (0.271 ± 0.051 vs. 0.185 ± 0.038) and partially discriminated pathological stages: M classification (*p* < 0.001) but not with stage, T, *n*, or CEA/CA125/CA19–9. ↑ m6A levels in the stage IV group (0.302 ± 0.063) than in stage I (0.243 ± 0.031), II (0.263 ± 0.031), or III groups (0.260 ± 0.048). ↑ in metastatic vs. non-metastatic CRC (0.302 ± 0.063 vs. 0.259 ± 0.041) and ↓ post-treatment. ROC-AUC m6A levels = 0.946 (95% CI 0.914–0.977), higher than CEA (0.817), CA125 (0.732), CA19–9 (0.771); combined model ↑ AUC = 0.977 (95% CI 0.961–0.994).	Monocytes identified as the most abundant m6A-modified immune cell type in PBMCs of CRC patients. m6A levels ↓ associated with monocyte immune response
GSE164191	NR	IGF2BP2 (m6A regulator) ↑ in CRC peripheral blood; Diagnostic performance: IGF2BP1 AUC = 0.710, IGF2BP2 AUC = 0.795, IGF2BP3 AUC = 0.710 (similar to CEA/CA125/CA19–9 but lower than m6A).	Monocytes showed ↑m6A levels in CRC. m6A writer/eraser/reader complexes positively correlated with monocyte infiltration ↑IGF2BP2 expression enriched in immune-related pathways: negative regulation of immune effector process, regulation of monocyte chemotaxis, cytokine production.
[15] Discovery set (Training and Validation set)	NR	↑m5C levels in PBMC CRC vs. controls (training: 0.383 ± 0.057 vs. 0.283 ± 0.058; validation: 0.373 ± 0.060). ↓ post-treatment (0.321 ± 0.045); ↑ with stage progression and + correlation with tumor tissue levels. m5C ↑ AUC than CEA (0.739), CA19–9 (0.669), CA125 (0.629); combined model vs. CEA/CA19–9/CA125 (AUC = 0.739/0.669/0.629); combined model ↑ AUC = 0.937 (95% CI 0.901–0.973). m5C and CEA independent diagnostic factors (m5C OR = 7.622) in the training set.	NR
GSE10715	NR	NR	↑ NSUN5, YBX1, TET2 (m5C regulators) in CRC blood immune cells. m5C complex + associated with monocyte infiltration
[11] Discovery set	NR	524 candidate biomarkers (CRC vs. controls PBMCs). 226/524 validated biomarkers (*p* < 0.001), 7 clusters defined CRC stages.	Functional enrichment: neutrophil-mediated immunity, platelet activation, wound healing, myeloid activation, chemotaxis in 524 candidate biomarkers In 226/524 validated biomarkers: early (I–II) ↑ cell cycle/B-cell activation, ↓ T-cell activation, ↑ myeloid migration; late (II–III) ↑ wound healing/coagulation, ROS metabolism, MPO.
GSE164541	NR	DEG clustering (*p* < 0.01) in adenoma/CRC vs. normal identified 6 expression patterns.	↓ T-cell activation/proliferation in adenomas and CRC; ↑ wound healing (CRC only), invasion (ECM organization), angiogenesis, and further suppression of adaptive immunity (IL-12 production). 11/226 shared concordant biomarkers (cross-comparison PBMCs–tumor): ↑ AQP9, GPR84, DUSP10, S100A8, S100A9, MCEMP1, CXCR1 and ↓NDRG2, EVL, TRAF3IP3, CD8 (myeloid activation, T-cell trafficking/activation).
6 GEOdatabases	NR	NR	Up/down patterns shared in 6/7 datasets (cross-comparison PBMCs–tumor) in 226 validated BMKs.
[10] Discovery set	Detection of 5 immunotypes (G1-G5): G1: naive T/B cells (mostly healthy); G2: memory CD4^+^ T + CD39^+^ Tregs; G3: NK + PD-1 ^+^/TIGIT^+^ CD8^+^ T cells; G4: NKT + TEM/TEMRA; G5: monocytes/neutrophils, ↓ lymphocytes.	Differences between healthy vs. cancer in RBCs, platelets, neutrophils, lymphocytes (not monocytes). Clustering separated healthy vs. cancer, not by diagnosis or therapy line. Cancer: ↑ CX3CR1^−^ CD8^+^ TEMRA and monocytes; Healthy: ↑ naive CD4^+^/CD8^+^ T cells and naive/memory B cells. Predictive model ROC-AUC = 0.91.	Functional signatures: G1–G2, TCF/LEF/CTNNB1, TCR, WNT/β-catenin; G4, cytotoxic T-cell response; G5, innate sensing/myeloid pathways. Cytokines: G1, FLT3LG, CCR7; G4, CCL4, TGFBR3; G5, CXCL16, IL1R1. TCR/BCR repertoire: G4: enriched dominant TCR clones (>10%), ↑ clonality (~3×), heterogeneous HLA overall. G1, highest BCR diversity.
GSE201085	↑ G5 in pathological complete response patients vs. Residual disease group. Immune shift with ↑ G3/G4 (aligned with G5) and ↓ G2 (aligned with G1), associated with chemotherapy response.	NR	NR
PDAC	No association between immunotype and PFS. G3-progressive associated with longer OS (*p* = 0.004). G3-based classifier discriminated long vs. short OS (ROC-AUC = 0.74). Chemo/Nivo: ↑ G3 in long vs. short OS (*p* = 0.0006), ROC-AUC = 0.94; ↓ G2-primed in long OS (ROC-AUC = 0.19). No significant effect in Chemo/Sotiga or Chemo/Nivo/Sotiga.		
HNSCC	HNSCC-Durva: ↑ G3-progressive scores with a trend for ↑ G4-chronic for responders at the on-treatment time point (*p* = 0.04), HNSCC-Nivo: Responders ↑ G2-primed immunotype (*p* = 0.004); predictive accuracy = 76% for responder vs. non-responder discrimination G4-chronic may stratify anti-PD-L1 responders independently of HPV status.		

Transcriptomic profiles of circulating immune cells are associated with CRC classification and correlate with classical tumour biomarkers and systemic inflammatory cell parameters across the six studies evaluated [10,11,12,13,14,15]. Abbreviations: AUC, area under the curve; BCR, B-cell receptor; BMKs, biomarkers; CA-125, cancer antigen 125; CA19–9, cancer antigen 19–9; CA72–4, cancer antigen 72–4; CCL2, C–C motif chemokine ligand 2; CCL4, C–C motif chemokine ligand 4; CCR7, C–C motif chemokine receptor 7; CEA, carcinoembryonic antigen; CI, confidence interval; COAD, colon adenocarcinoma; CRP, C-reactive protein; CRC, colorectal cancer; CSS, cancer-specific survival; CXCL16, C–X–C motif chemokine ligand 16; DFS, disease-free survival; ECM, extracellular matrix; ESR, erythrocyte sedimentation rate; FABP4, fatty acid-binding protein 4; FLT3LG, Fms-related tyrosine kinase 3 ligand; GO, Gene Ontology; HLA-DR, human leukocyte antigen–DR isotype; IFN-γ, interferon gamma; IGF2BP, insulin-like growth factor 2 mRNA-binding protein; IL, interleukin; m5C, 5-methylcytosine; m6A, N6-methyladenosine; mGPS, modified Glasgow prognostic score; MPO, myeloperoxidase; NAC, neoadjuvant chemotherapy; NK, natural killer; NKT, natural killer T cells; NR, not reported; NSUN5, NOP2/Sun RNA methyltransferase family member 5; OR, odds ratio; OS, overall survival; PBMCs, peripheral blood mononuclear cells; PD-1, programmed cell death protein 1; PD-L1, programmed death-ligand 1; PFS, progression-free survival; ROC, receiver operating characteristic; TAMs, tumor-associated macrophages; TCR, T-cell receptor; TEM, effector memory T cells; TEMRA, terminally differentiated effector memory T cells re-expressing CD45RA; TGFBR3, transforming growth factor beta receptor 3; TIGIT, T cell immunoreceptor with Ig and ITIM domains; TNF-α, tumor necrosis factor alpha; WNT, wingless-related integration site signaling pathway. Arrows indicate direction of gene regulation: ↑ upregulation; ↓ downregulation.

## Data Availability

No new data were created in this study. Data sharing is not applicable to this article.

## Supplementary Materials

The following supporting information can be downloaded at: https://www.mdpi.com/article/10.3390/ijms27094143/s1.
