# Supplementary material for: Systematic Review of Monocyte Transcriptomic Profiles as Diagnostic and Prognostic Biomarkers in Colorectal Cancer
Source: Int J Mol Sci. 2026 May 6;27(9):4143. doi: 10.3390/ijms27094143 (PMC13163292; doi:10.3390/ijms27094143)
Supplement: Supplementary file 1 [file ijms-27-04143-s001.zip › Supplementary Materials_Figure S1.pptx]

## Slide 1
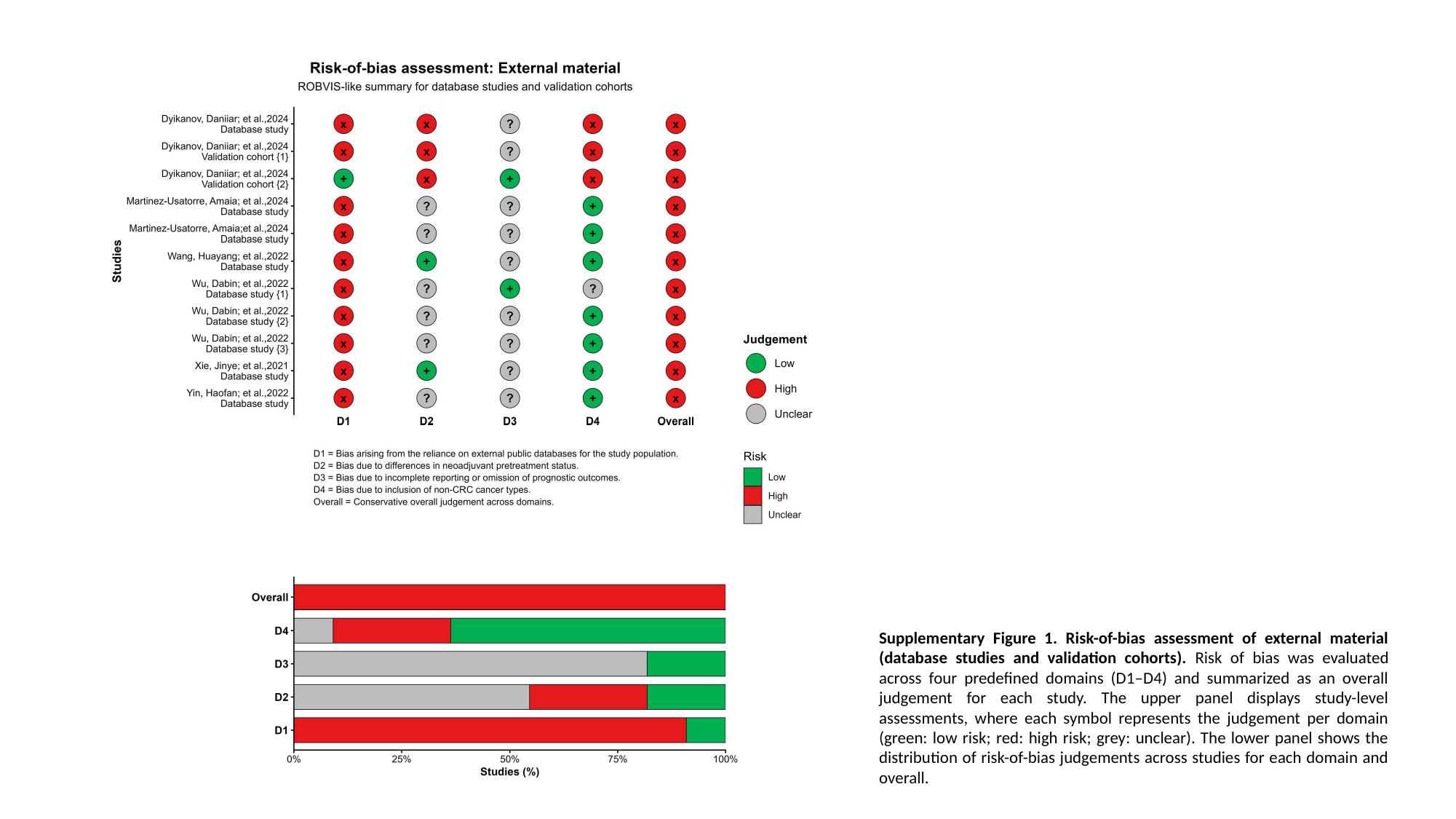

Supplementary Figure 1. Risk-of-bias assessment of external material (database studies and validation cohorts). Risk of bias was evaluated across four predefined domains (D1–D4) and summarized as an overall judgement for each study. The upper panel displays study-level assessments, where each symbol represents the judgement per domain (green: low risk; red: high risk; grey: unclear). The lower panel shows the distribution of risk-of-bias judgements across studies for each domain and overall.
