# Supplementary material for: Systematic Review of Monocyte Transcriptomic Profiles as Diagnostic and Prognostic Biomarkers in Colorectal Cancer
Source: Int J Mol Sci. 2026 May 6;27(9):4143. doi: 10.3390/ijms27094143 (PMC13163292; doi:10.3390/ijms27094143)
Supplement: Supplementary file 1 [file ijms-27-04143-s001.zip › Supplementary Materials_Tables2.pdf]

Supplementary Materials

Table S2. Baseline Search Formulas for Our Databases: PubMed, Scopus, Web of Science, and Embase **(2019-2024)**

Database	Search Query
PubMed	(((Colorectal cancer OR Colon cancer OR Rectal cancer OR CRC OR Colorectal neoplasm) AND<br>(Monocytes OR Circulating monocytes OR Monocyte precursors)) AND<br>(Transcriptomic biomarkers OR Transcriptomic OR Transcriptomic profile OR Gene expression OR RNA-Seq) AND<br>(Biomarker OR Marker OR Predictive biomarker OR Prognostic biomarker OR Diagnostic biomarker) AND<br>(Stage OR Survival OR Prognosis OR Disease-free survival OR Overall survival OR Recurrence-free survival OR Treatment response OR Therapy response OR Disease diagnosis OR Disease prognosis OR 5-year survival rate))
Scopus	((colorectal AND cancer OR colon AND cancer OR rectal AND cancer OR crc OR colorectal AND neoplasm) AND<br>(monocytes OR circulating AND monocytes OR monocyte AND precursors) AND<br>(transcriptomic OR gene AND expression OR RNA-seq) AND<br>(biomarker OR marker OR predictive AND biomarker OR prognostic AND biomarker OR diagnostic AND biomarker) AND<br>(survival OR prognosis OR disease-free AND survival OR overall AND survival OR recurrence-free AND survival OR treatment AND response OR therapy AND response OR disease AND diagnosis OR disease AND prognosis OR 5-year AND survival AND rate))
Web of Science	TS = ((Colorectal cancer OR Colon cancer OR Rectal cancer OR CRC OR Colorectal neoplasm) AND<br>(Monocytes OR Circulating monocytes OR Monocyte precursors) AND<br>(Transcriptomic biomarkers OR Transcriptomic OR Transcriptomic profile OR Gene expression OR RNA-Seq) AND<br>(Biomarker OR Marker OR Predictive biomarker OR Prognostic biomarker OR Diagnostic biomarker) AND<br>(Stage OR Survival OR Prognosis OR Disease-free survival OR Overall survival OR Recurrence-free survival OR Treatment response OR Therapy response OR Disease diagnosis OR Disease prognosis OR 5-year survival rate))
Embase	('colorectal cancer':ti,ab OR 'colon cancer':ti,ab OR 'rectal cancer':ti,ab OR 'crc':ti,ab OR 'colorectal neoplasm':ti,ab) AND<br>('monocytes':ti, ab OR 'circulating monocytes':ti, ab OR 'monocyte precursors':ti, ab) AND<br>('transcriptomic' OR 'gene expression' OR 'rna-seq') AND<br>('biomarker' OR 'marker' OR 'predictive biomarker' OR 'prognostic biomarker' OR 'diagnostic biomarker') AND<br>('survival' OR 'prognosis' OR 'disease-free survival' OR 'overall survival' OR 'recurrence-free survival' OR 'treatment response' OR 'therapy response' OR 'disease diagnosis' OR 'disease prognosis' OR '5-year survival rate')
